# Supplementary material for: Social memory deficit caused by dysregulation of the cerebellar vermis
Source: Nat Commun. 2023 Sep 26;14:6007. doi: 10.1038/s41467-023-41744-2 (PMC10522595; doi:10.1038/s41467-023-41744-2)
Supplement: Supplementary file 1 — Supplementary Information [file 41467_2023_41744_MOESM1_ESM.pdf]

Supplementary Information

Table of Contents

Supplementary Figures

Supplementary Fig. 1 Expression of the chemogenetic receptor in the cerebellum.

Supplementary Fig. 2 Chemogenetic excitation of MLIs in cerebellar lobules IV-VII did not affect anxiety levels, locomotor activity, motor coordination or free social interaction.

Supplementary Fig. 3 Effects of chemogenetic excitation of MLIs in cerebellar lobules IV-VII on c-Fos expression in different brain regions after the social recognition test.

Supplementary Fig. 4 Subtypes of c-Fos positive neurons in the key brain regions of social recognition network.

Supplementary Fig. 5 Anterograde tracing of the fastigial nucleus outputs.

Supplementary Fig. 6 Retrograde tracing of the basolateral amygdala inputs.

Supplementary Fig. 7 Integration of the FN outputs and BLA inputs in the social memory network.

Supplementary Fig. 8 Example images of the prelimbic cortex (PL) from three individual subjects.

Supplementary Tables

Supplementary Table 1 A list of statistical analyses.

The supplementary information is for:

Social memory deficit caused by dysregulation of the cerebellar vermis.

Owen Y Chao, Salil Saurav Pathak, Hao Zhang, George J Augustine, Jason M Christie, Chikako Kikuchi, Hiroki Taniguchi, Yi-Mei Yang*

* Corresponding author

Yi-Mei Yang, PhD

Department of Biomedical Sciences

University of Minnesota Medical School

1035 University Drive

Duluth MN 55812, USA

Phone: +1 218-726-7818

Email: ymyang@d.umn.edu

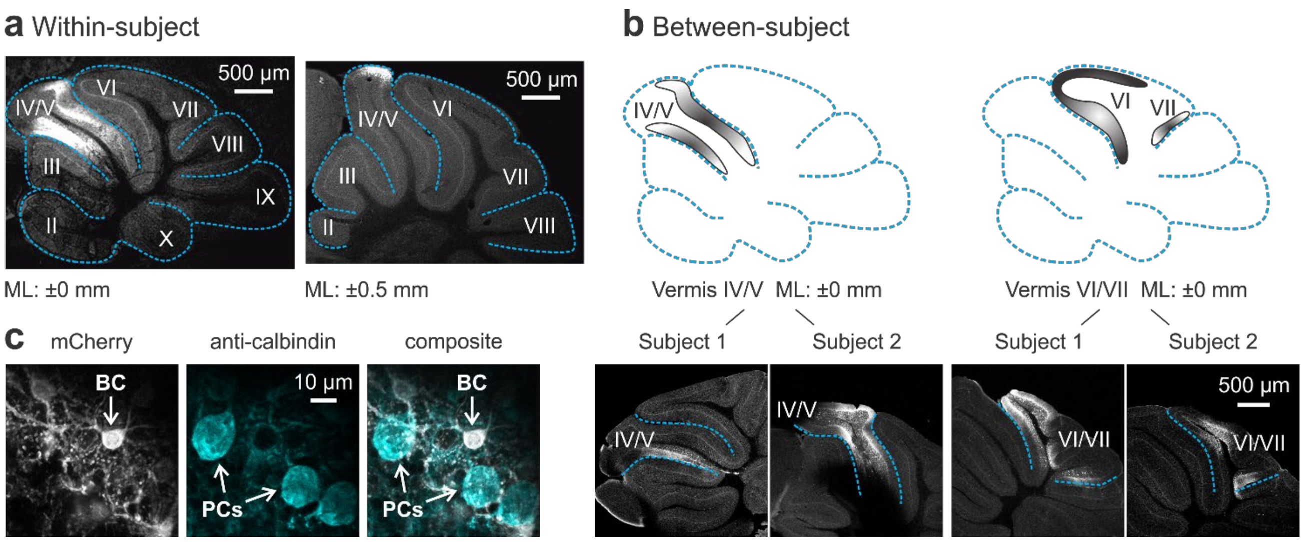

Supplementary Fig. 1 Expression of the chemogenetic receptor in the cerebellum. **a** Cerebellar slices from a c-kit$^{IRES\text{-}Cre}$ mouse injected with AAV8-hSyn-DIO-hM3Dq-mCherry in the anterior vermis showing expression of the hM3Dq receptor (white) in a midsagittal section (left) and in a section 0.5 mm away from the midline (right). ML, mediolateral. **b** Schematic drawing of the hM3Dq distribution (gray areas) in midsagittal sections across different mice (n=6) in the vermis IV/V (left) or VI/VII (right) group. Two individual examples for each group are shown below. **c** Confocal images of the hM3Dq expression (visualized with mCherry) in a basket cell (BC). Co-labeling of Purkinje cells (PCs) using an anti-Calbindin antibody showed that the BC formed synapses onto four PCs. The images represented average fluorescent intensity in cerebellar sections from 6 mice.

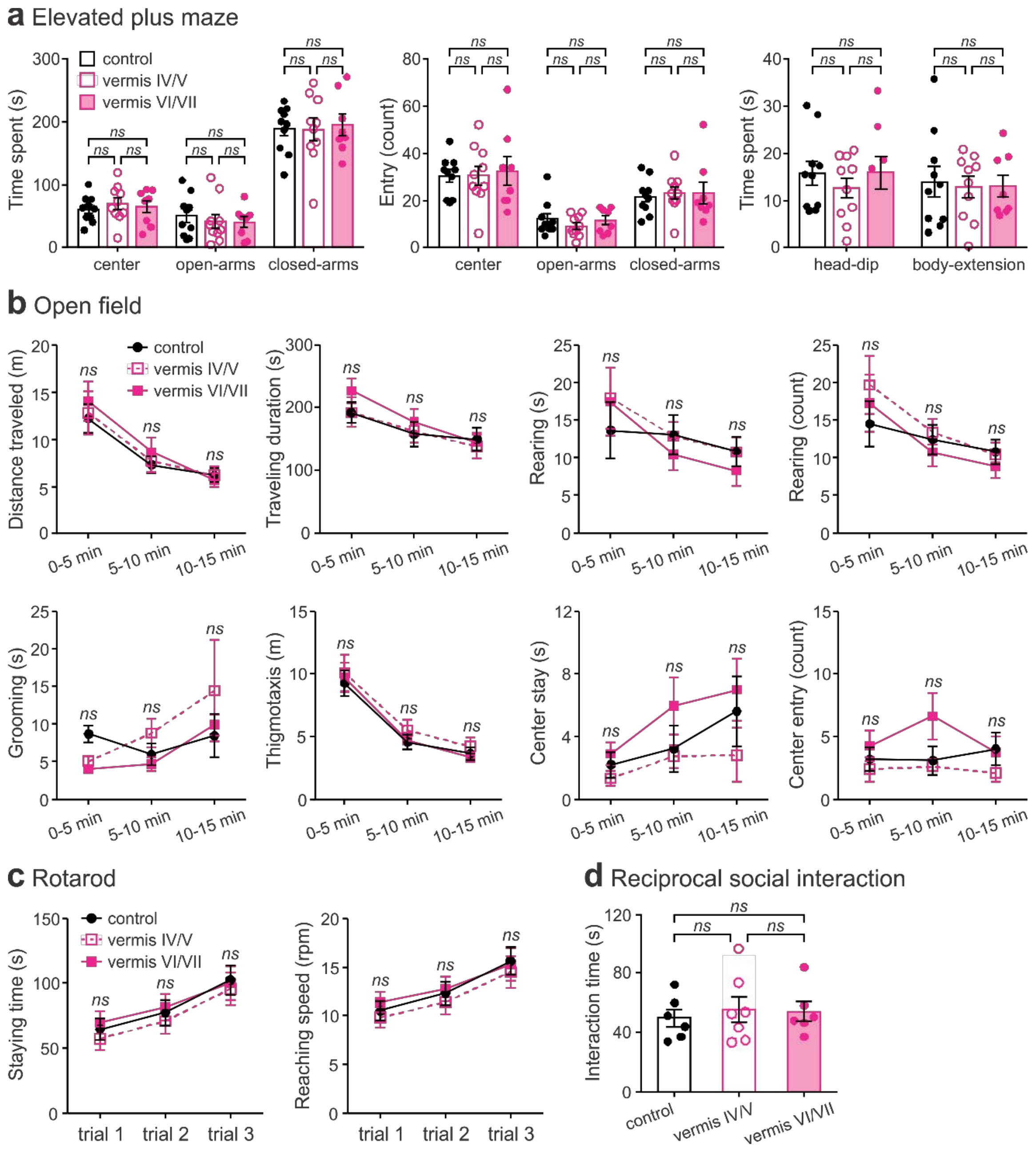

Supplementary Fig. 2 Chemogenetic excitation of MLIs in cerebellar lobules IV-VII did not affect anxiety levels, locomotor activity, motor coordination or free social interaction. **a-d** No differences were found between control, vermis IV/V, and vermis VI/VII groups in behavioral parameters obtained from elevated plus maze (control: n=10 mice, vermis IV/V: n=10 mice, vermis VI/VII: n=8 mice; **a**), open field (control: n=10 mice, vermis IV/V: n=10 mice, vermis VI/VII: n=8 mice; **b**), rotarod (control: n=10 mice, vermis IV/V: n=10 mice, vermis VI/VII: n=8 mice; **c**) and reciprocal social interaction (control: n=6 mice, vermis IV/V: n=7 mice, vermis VI/VII: n=6; **d**) tests. One-way or two-way ANOVA was used for group comparisons. *ns*, not significant. Data are presented as the mean ± SEM and the center of error bars is the mean. Source data are provided as a Source Data file.

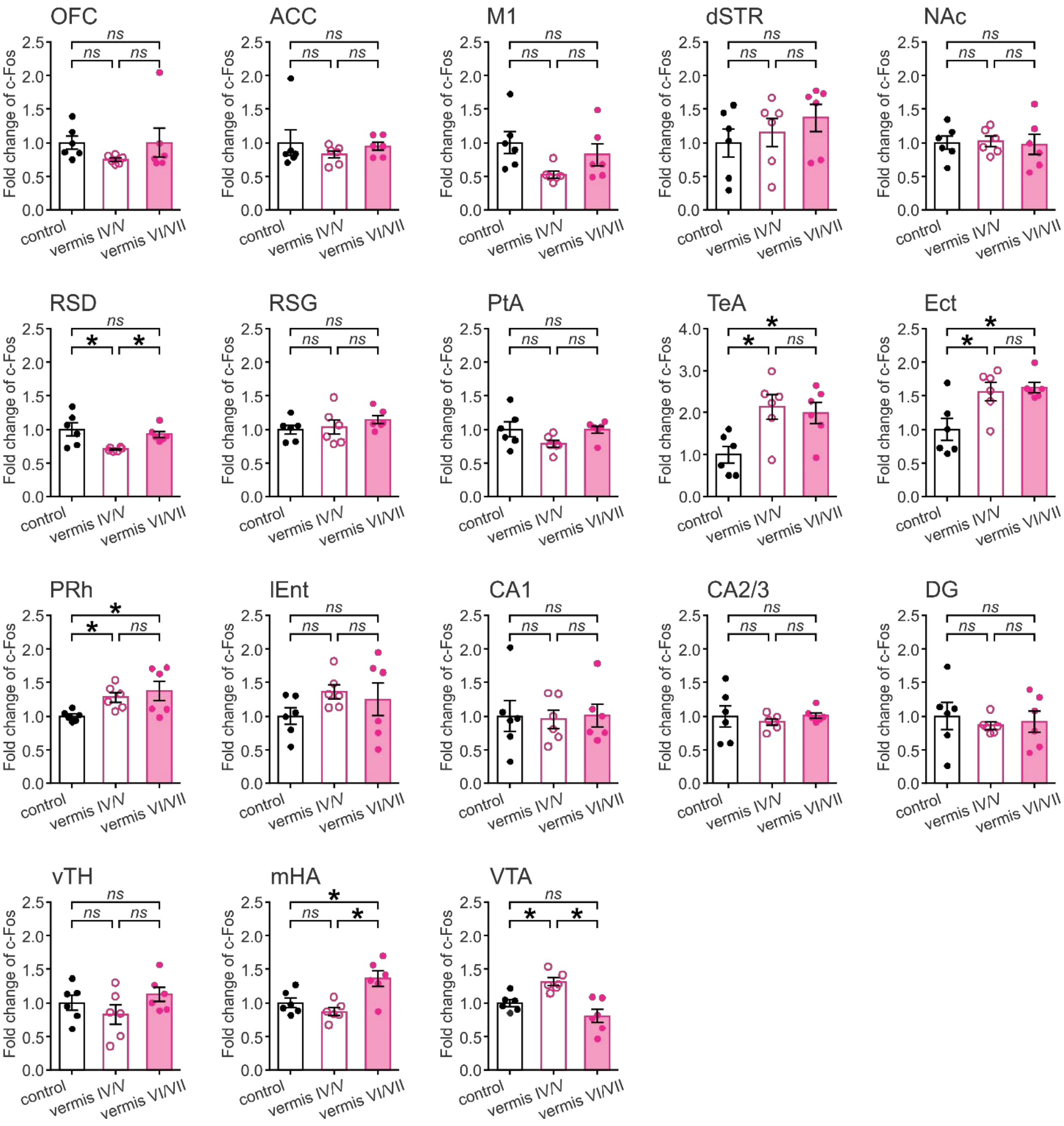

Supplementary Fig. 3 Effects of chemogenetic excitation of MLIs in cerebellar lobules IV-VII on c-Fos expression in different brain regions after the social recognition test. Fold change, defined as the number of c-Fos-positive cells in each group divided by the average value of the control group, was summarized for all regions. *p<0.05, two-tailed Fisher's LSD test. *ns*, not significant. n=6 mice/group. Data are presented as the mean ± SEM and the center of error bars is the mean. Source data are provided as a Source Data file. OFC, orbitofrontal cortex; ACC, anterior cingulate cortex; M1, primary motor cortex; dSTR, dorsal striatum; NAc, nucleus accumbens; RSD, retrosplenial dysgranular cortex; RSG, retrosplenial granular cortex; PtA, parietal association cortex; TeA, temporal association cortex; Ect, ectorhinal cortex; PRh, perirhinal cortex; lEnt, lateral entorhinal cortex; CA1, hippocampus CA1; CA2/3, hippocampus CA2/3; DG, dentate gyrus; vTH, ventral thalamus; mHA, medial hypothalamus; VTA, ventral tegmental area.

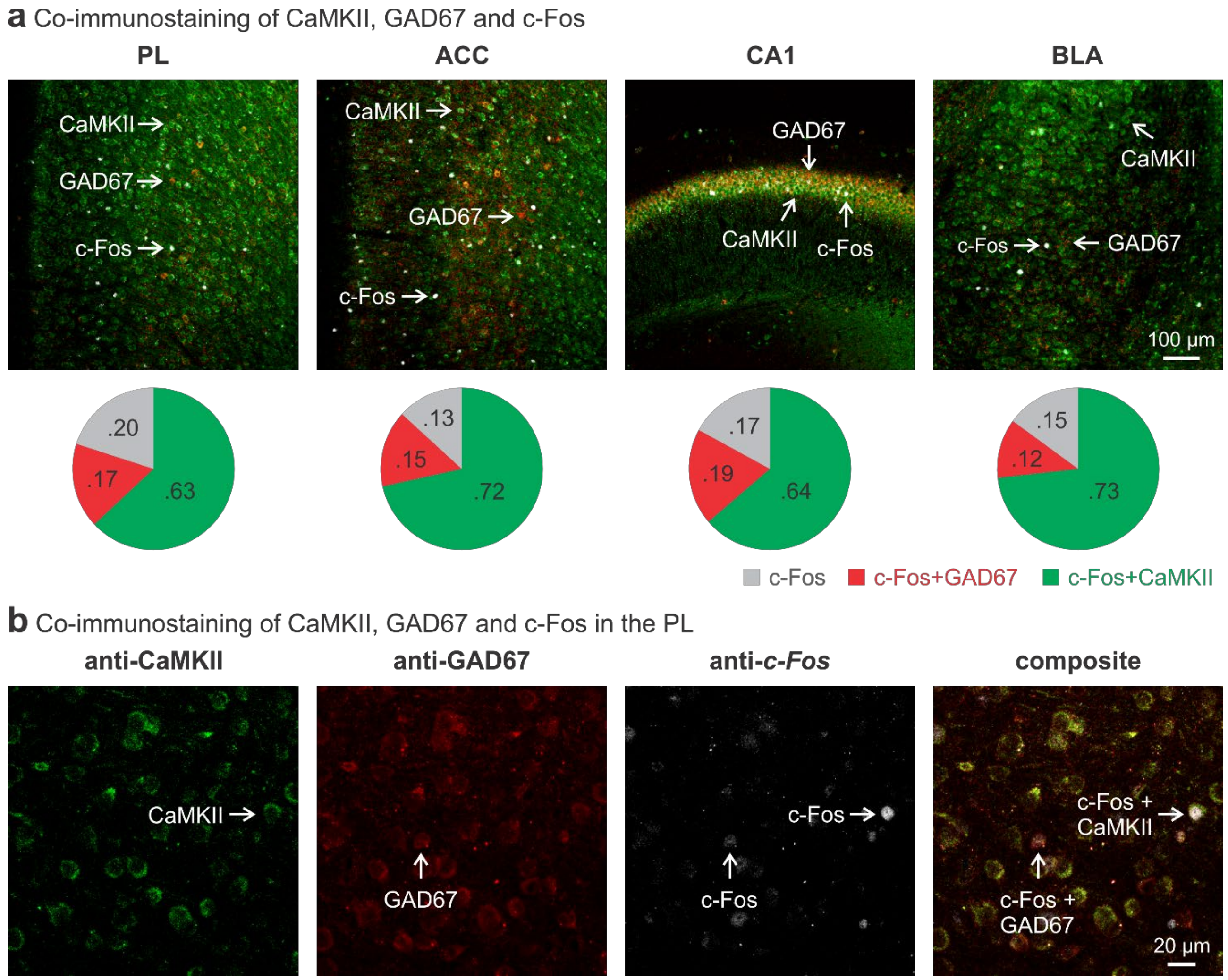

Supplementary Fig. 4 Subtypes of c-Fos positive neurons in the key brain regions of social recognition network. **a** Confocal images of co-immunostaining c-Fos (a marker of neuronal activity; white), CaMKII (a marker of glutamatergic neurons; green), and GAD67 (a marker of GABAergic neurons; red) in the prelimbic cortex (PL), anterior cingulate cortex (ACC), hippocampus CA1 (CA1), and basolateral amygdala (BLA) following the social recognition test. Percentage of neurons containing c-Fos only, c-Fos and CaMKII, or c-Fos and GAD67 were summarized below for each region (n=6 samples). **b** Higher-magnification images of co-staining CaMKII, GAD67, and c-Fos in the PL. The images represented average fluorescent intensity in each region from 6 samples. Source data are provided as a Source Data file.

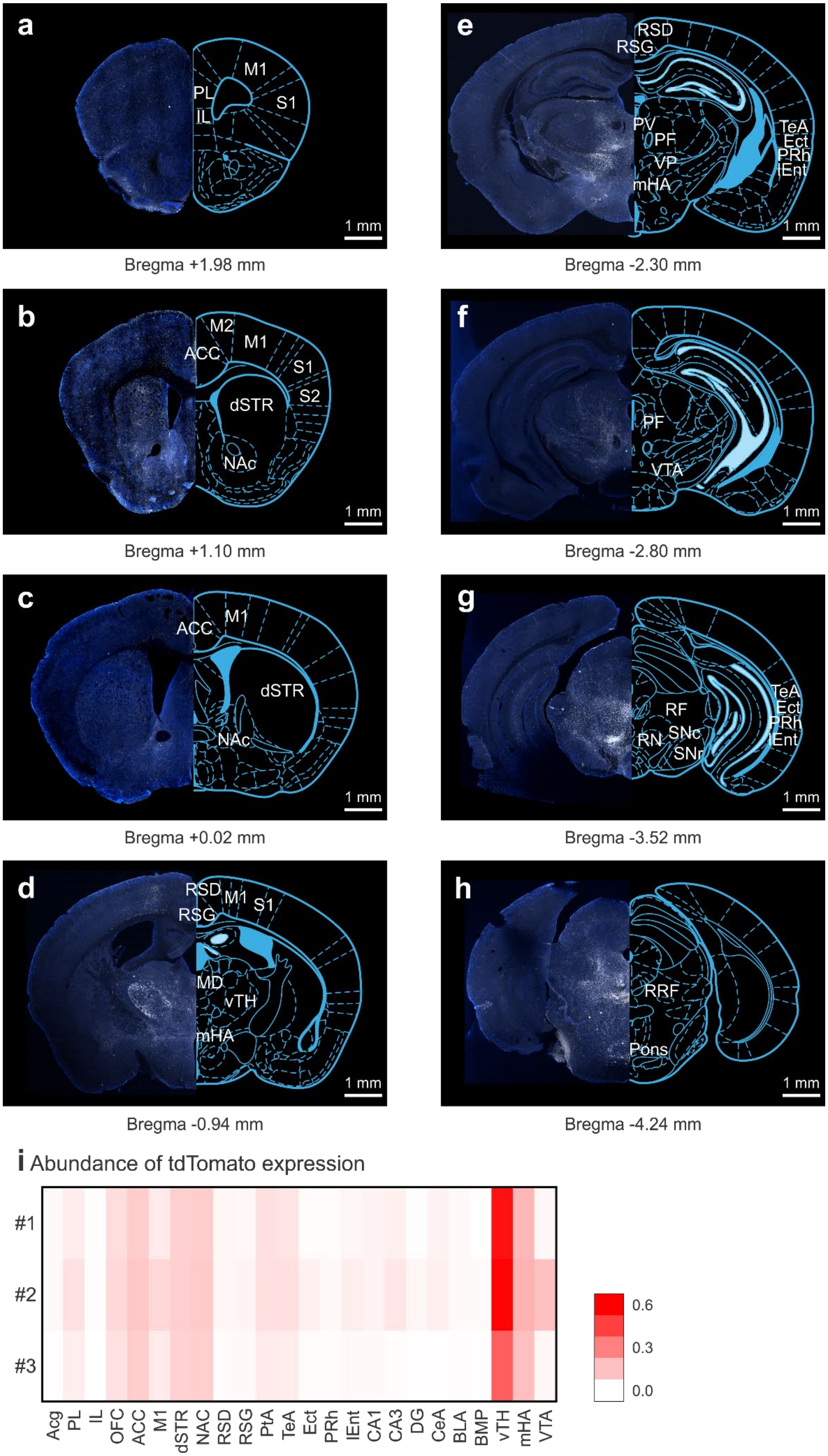
a
M1
PL
S1
IL
1 mm
Bregma +1.98 mm
b
M2
M1
ACC
S1
dSTR
S2
NAc
1 mm
Bregma +1.10 mm
c
M1
ACC
dSTR
NAc
1 mm
Bregma +0.02 mm
d
RSD
M1
S1
RSG
MD
vTH
mHA
1 mm
Bregma -0.94 mm
e
RSD
RSG
PV
PF
VP
mHA
TeA
Ect
PRh
lEnt
1 mm
Bregma -2.30 mm
f
PF
VTA
1 mm
Bregma -2.80 mm
g
TeA
Ect
PRh
lEnt
RF
RN
SNc
SNr
1 mm
Bregma -3.52 mm
h
RRF
Pons
1 mm
Bregma -4.24 mm
i Abundance of tdTomato expression
#1
#2
#3
0.6
0.3
0.0
Acg
PL
IL
OFC
ACC
M1
dSTR
NAC
RSD
RSG
PtA
TeA
Ect
PRh
lEnt
CA1
CA3
DG
CeA
BLA
BMP
vTH
mHA
VTA

Supplementary Fig. 5 Anterograde tracing of the fastigial nucleus outputs. **a-h** Coronal sections from Ai9 mice (n=3) infused with AAV1-hSyn-Cre in the fastigial nucleus as illustrated in **Fig. 6**. Neurons in the fastigial nucleus and neurons that received mono- or disynaptic input from this region were labeled with tdTomato (white). Cell nuclei were stained with DAPI (blue). Brain regions that contained tdTomato-expressing neurons and/or their processes were indicated. All sections were manually overlaid onto the mouse brain atlas. The atlas was adapted from *Franklin, K. B. J. & Paxinos, G. The mouse brain in stereotaxic coordinates, compact third edition (Academic Press; 3rd edition, 2008)*. **i** Summary of tdTomato labeling in social recognition-relevant 24 brain regions (n=2-3 sections/region) across three Ai9 mice. The abundance of tdTomato expression was calculated by normalizing the fluorescence intensity to the background in each region. The images represented average fluorescent intensity in each region from 3 mice. Source data are provided as a Source Data file. ACC, anterior cingulate cortex; Acg, anterior cingulate cortex (rostral); BLA, basolateral amygdala; BMP, basomedial amygdala; CA1, hippocampus CA1; CA2/3, hippocampus CA2/3; CeA, central nucleus of amygdala; DG, dentate gyrus; dSTR, dorsal striatum; Ect, ectorhinal cortex; IL, infralimbic cortex; lEnt, lateral entorhinal cortex; M1, primary motor cortex; M2, secondary motor cortex; MD, mediodorsal thalamus; mHA, medial hypothalamus; NAc, nucleus accumbens; OFC, orbitofrontal cortex; PF, parafascicular thalamus; PL, prelimbic cortex; PRh, perirhinal cortex; PtA, parietal association cortex; PV, paraventricular thalamus; RF, reticular formation; RN, red nucleus; RRF, retrorubral field; RSD, retrosplenial dysgranular cortex; RSG, retrosplenial granular cortex; S1, primary somatosensory cortex; S2, secondary somatosensory cortex; SNc, substantia nigra pars compacta; SNr, substantia nigra pars reticulata; TeA, temporal association cortex; VP, ventroposterior thalamus; VTA, ventral tegmental area; vTH, ventral thalamus.

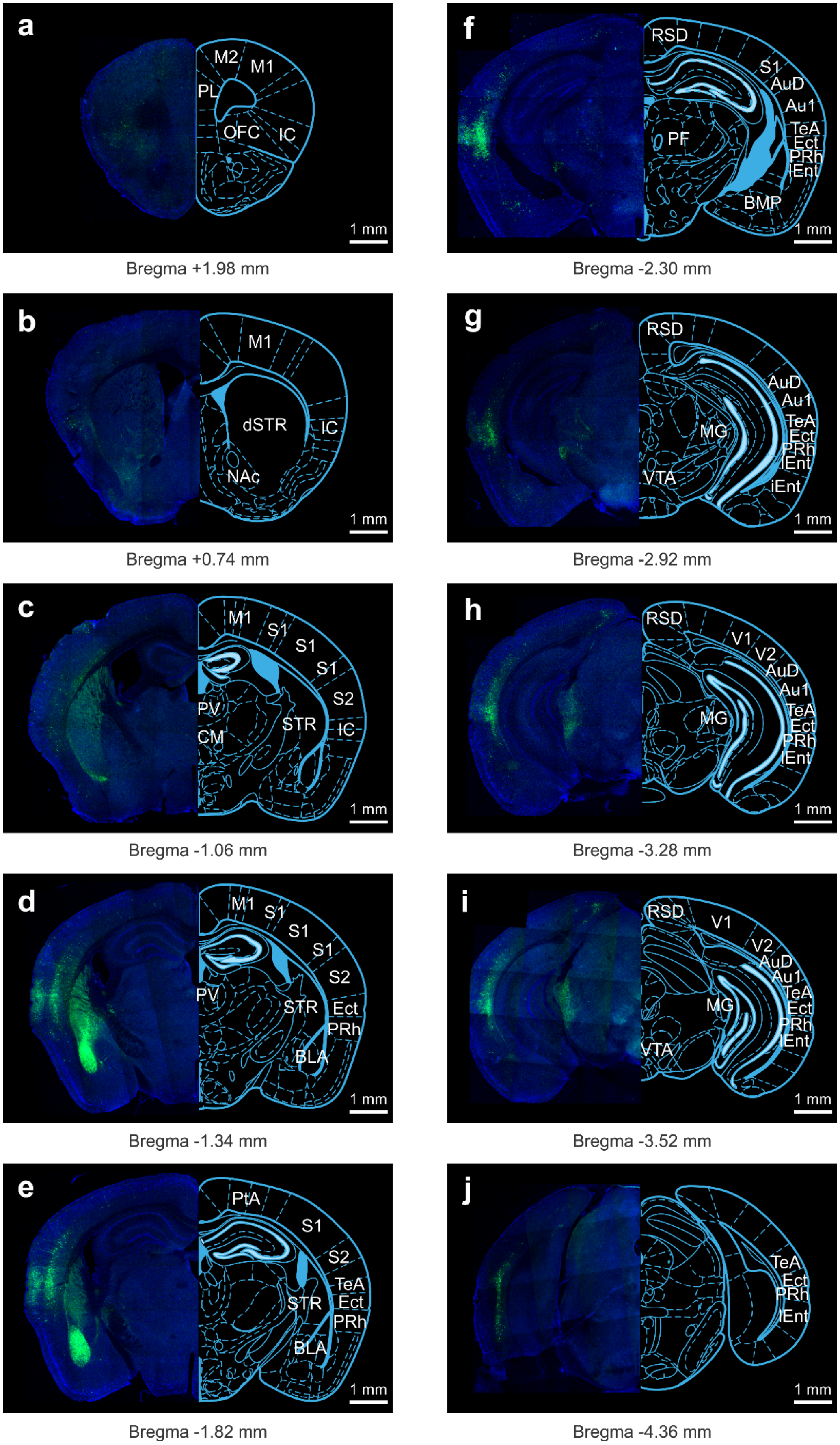
a
M2
M1
PL
OFC
IC
1 mm
Bregma +1.98 mm
b
M1
dSTR
IC
NAc
1 mm
Bregma +0.74 mm
c
M1
S1
S1
S1
S2
PV
STR
IC
CM
1 mm
Bregma -1.06 mm
d
M1
S1
S1
S1
S2
PV
STR
Ect
PRh
BLA
1 mm
Bregma -1.34 mm
e
PtA
S1
S2
TeA
STR
Ect
PRh
BLA
1 mm
Bregma -1.82 mm
f
RSD
S1
AuD
Au1
TeA
Ect
PRh
lEnt
PF
BMP
1 mm
Bregma -2.30 mm
g
RSD
AuD
Au1
TeA
Ect
PRh
lEnt
MG
VTA
iEnt
1 mm
Bregma -2.92 mm
h
RSD
V1
V2
AuD
Au1
TeA
Ect
PRh
lEnt
MG
1 mm
Bregma -3.28 mm
i
RSD
V1
V2
AuD
Au1
TeA
Ect
PRh
lEnt
MG
VTA
1 mm
Bregma -3.52 mm
j
TeA
Ect
PRh
lEnt
1 mm
Bregma -4.36 mm

Supplementary Fig. 6 Retrograde tracing of the basolateral amygdala inputs. **a-j** Coronal sections from Ai9 mice (n=3) infused with AAVrg-hSyn-EGFP in the basolateral amygdala as illustrated in **Fig. 8**. Neurons in the basolateral amygdala and neurons that provided direct input to this region were labeled with EGFP (green). Cell nuclei were stained with DAPI (blue). Brain regions that contained EGFP-expressing neurons and/or their processes were indicated. The images represented average fluorescent intensity in each region from 3 mice. All sections were manually overlaid onto the mouse brain atlas. The atlas was adapted from *Franklin, K. B. J. & Paxinos, G. The mouse brain in stereotaxic coordinates, compact third edition (Academic Press; 3rd edition, 2008)*. Au1, primary auditory cortex; AuD, secondary auditory cortex; BLA, basolateral amygdala; BMP, basomedial amygdala; dSTR, dorsal striatum; Ect, ectorhinal cortex; IC, insular cortex; lEnt, lateral entorhinal cortex; M1, primary motor cortex; M2, secondary motor cortex; MG; medial geniculate nucleus; NAc, nucleus accumbens; PL, prelimbic cortex; OFC, orbitofrontal cortex; PF, parafascicular thalamus; PRh, perirhinal cortex; PtA, parietal association cortex; PV, paraventricular thalamus; RSD, retrosplenial dysgranular cortex; S1, primary somatosensory cortex; S2, secondary somatosensory cortex; TeA, temporal association cortex; V1, primary visual cortex; V2, secondary visual cortex; VTA, ventral tegmental area.

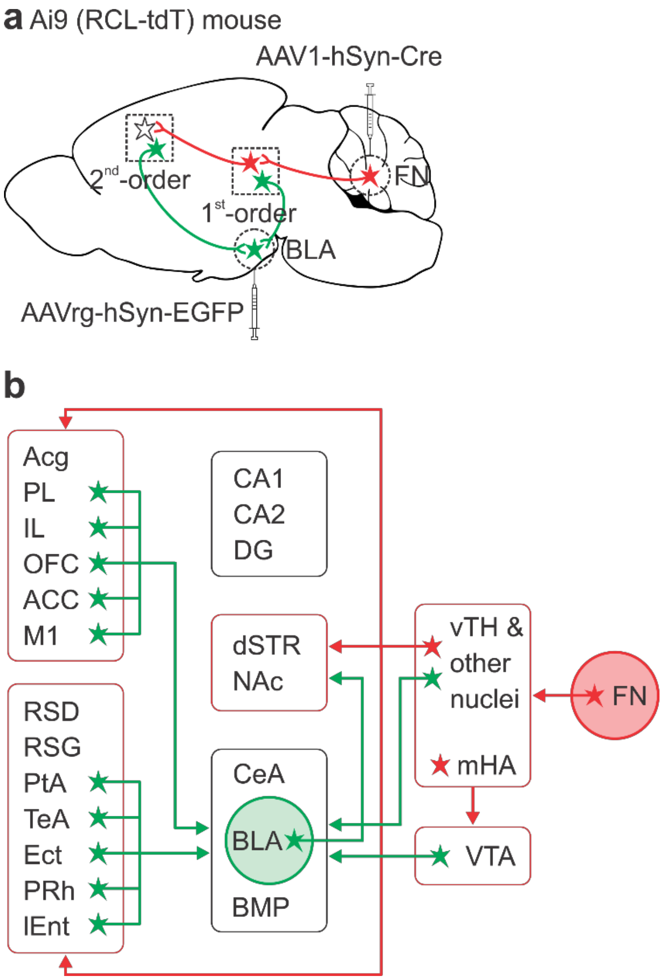

Supplementary Fig. 7 Integration of the FN outputs and BLA inputs in the social memory network. **a** Strategy for anterograde tracing of the fastigial nucleus (FN) outputs with AAV1-hSyn-Cre and retrograde tracing of the basolateral amygdala (BLA) inputs with AAVrg-hSyn-EGFP in Ai9 mice as shown in **Fig. 8**. **b** Summary of the major FN outputs and BLA inputs in 24 brain regions underlying social recognition memory. FN and its downstream neurons are indicated in red. BLA and its upstream neurons are indicated in green. Note the information flow from the FN (right) to first-order nuclei (mainly vTH and other thalamic nuclei) to second-order nuclei in the subcortex and cortex (left). The BLA is integrated in the network via monosynaptic connections with multiple brain regions. ACC, anterior cingulate cortex; Acg, anterior cingulate cortex (rostral); BLA, basolateral amygdala; BMP, basomedial amygdala; CA1, hippocampus CA1; CA2/3, hippocampus CA2/3; CeA, central nucleus of amygdala; DG, dentate gyrus; dSTR, dorsal striatum; Ect, ectorhinal cortex; IL, infralimbic cortex; lEnt, lateral entorhinal cortex; M1, primary motor cortex; mHA, medial hypothalamus; NAc, nucleus accumbens; OFC, orbitofrontal cortex; PL, prelimbic cortex; PRh, perirhinal cortex; PtA, parietal association cortex; RSD, retrosplenial dysgranular cortex; RSG, retrosplenial granular cortex; TeA, temporal association cortex; VTA, ventral tegmental area; vTH, ventral thalamus.

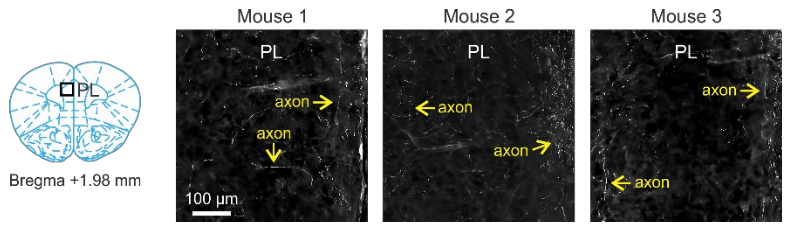

Supplementary Fig. 8 Example images of the prelimbic cortex (PL) from three individual subjects. The axons (white) were labeled via infusion of AAV1-hSyn-Cre in the fastigial nucleus of Ai9 mice (n=3), as illustrated in **Fig. 6**. The PL was indicated with the mouse brain atlas (left). The atlas was adapted from *Franklin, K. B. J. & Paxinos, G. The mouse brain in stereotaxic coordinates, compact third edition (Academic Press; 3rd edition, 2008)*.

Supplementary Table 1: A list of statistical analyses.

Chemogenetic manipulation – electrophysiology	
Mixed two-way ANOVA<br>hM3Dq (+) vs hM3Dq (-) (MLIs)<br>baseline vs CNO (MLIs)	"group" effect: $F_{1,12}$=2.065, *p*>0.05<br>"CNO" effect: $F_{1,12}$=18.86, ***p*=0.001**<br>"group x CNO" effect: $F_{1,12}$=13.02, ***p*=0.004**
Paired *t*-test within group<br>baseline vs CNO (MLIs)	hM3Dq (+): t_4=4.488, ***p*=0.011**<br>hM3Dq (-): t_5=-0.04, *p*>0.05
Independent *t*-test between groups<br>hM3Dq (+) vs hM3Dq (-) (MLIs)	baseline: t_9=0.8499, *p*>0.05<br>CNO: t_9=2.626, ***p*=0.028**
Mixed two-way ANOVA<br>hM3Dq (+) vs hM3Dq (-) (PCs-frequency)<br>baseline vs CNO (PCs-frequency)	"group" effect: $F_{1,9}$=3.522, *p*>0.05<br>"CNO" effect: $F_{1,9}$=20, ***p*=0.002**<br>"group x CNO" effect: $F_{1,9}$=20.28, ***p*=0.002**
Paired *t*-test within group<br>baseline vs CNO (PCs-frequency)	hM3Dq (+): t_6=4.272, ***p*=0.005**<br>hM3Dq (-): t_6=-1.004, *p*>0.05
Independent *t*-test between groups<br>hM3Dq (+) vs hM3Dq (-) (PCs-frequency)	baseline: t_{12}=0.05175, *p*>0.05<br>CNO: t_{12}=2.808, ***p*=0.016**
Mixed two-way ANOVA<br>hM3Dq (+) vs hM3Dq (-) (PCs-CV)<br>baseline vs CNO (PCs-CV)	"group" effect: $F_{1,12}$=4.631, *p*>0.05<br>"CNO" effect: $F_{1,12}$=6.87, ***p*=0.022**<br>"group x CNO" effect: $F_{1,12}$=8.836, ***p*=0.011**
Paired *t*-test within group<br>baseline vs CNO (PCs-CV)	hM3Dq (+): t_6=2.814, ***p*=0.03**<br>hM3Dq (-): t_6=-1.575, *p*>0.05
Independent *t*-test between groups<br>hM3Dq (+) vs hM3Dq (-) (PCs-CV)	baseline: t_{12}=1.299, *p*>0.05<br>CNO: t_{12}=2.542, ***p*=0.026**
**Chemogenetic manipulation – behavioral tests**	
Elevated plus maze	
One-way ANOVA	center time: "group" effect, $F_{2,25}$=0.3, *p*>0.05<br>open-arms time: "group" effect, $F_{2,25}$=0.262, *p*>0.05<br>closed-arms time: "group" effect, $F_{2,25}$=0.053, *p*>0.05<br>center entry: "group" effect, $F_{2,25}$=0.072, *p*>0.05<br>open-arms entry: "group" effect, $F_{2,25}$=0.74, *p*>0.05<br>closed-arms entry: "group" effect, $F_{2,25}$=0.091, *p*>0.05<br>head-dips time: "group" effect, $F_{2,25}$=0.478, *p*>0.05<br>body-extension time: "group" effect, $F_{2,25}$=0.056, *p*>0.05<br>distance traveled: "group" effect, $F_{2,25}$=0.443, *p*>0.05
Open field	
Mixed two-way ANOVA<br>(Distance traveled)	"group" effect: $F_{2,25}$=0.152, *p*>0.05<br>"interval" effect: $F_{2,50}$=50.193, ***p*<0.001**<br>"group x interval" effect: $F_{4,50}$=0.461, *p*>0.05
Mixed two-way ANOVA<br>(Traveling duration)	"group" effect: $F_{2,25}$=0.252, *p*>0.05<br>"interval" effect: $F_{2,50}$=31.331, ***p*<0.001**<br>"group x interval" effect: $F_{4,50}$=1.358, *p*>0.05
Mixed two-way ANOVA<br>(Rearing duration)	"group" effect: $F_{2,25}$=0.159, *p*>0.05<br>"interval" effect: $F_{2,50}$=7.429, ***p*=0.001**<br>"group x interval" effect: $F_{4,50}$=0.84, *p*>0.05
Mixed two-way ANOVA	"group" effect: $F_{2,25}$=0.367, *p*>0.05

(Rearing count)	"interval" effect: $F_{2,50}$=11.551, ***p*<0.001**<br>"group x interval" effect: $F_{4,50}$=0.797, *p*>0.05
Mixed two-way ANOVA<br>(Grooming)	"group" effect: $F_{2,25}$=1.218, *p*>0.05<br>"interval" effect: $F_{2,50}$=2.494, *p*>0.05<br>"group x interval" effect: $F_{4,50}$=0.736, *p*>0.05
Mixed two-way ANOVA<br>(Thigmotaxis)	"group" effect: $F_{2,25}$=0.352, *p*>0.05<br>"interval" effect: $F_{2,50}$=70.223, ***p*<0.001**<br>"group x interval" effect: $F_{4,50}$=0.11, *p*>0.05
Mixed two-way ANOVA<br>(Center stay)	"group" effect: $F_{2,25}$=1.416, *p*>0.05<br>"interval" effect: $F_{2,50}$=7.138, ***p*=0.002**<br>"group x interval" effect: $F_{4,50}$=0.757, *p*>0.05
Mixed two-way ANOVA<br>(Center entry)	"group" effect: $F_{2,25}$=1.453, *p*>0.05<br>"interval" effect: $F_{2,50}$=2.111, *p*>0.05<br>"group x interval" effect: $F_{4,50}$=2.925, ***p*=0.03**
One-way ANOVA<br>(Distance traveled)	0-5 min: "group" effect, $F_{2,25}$=0.226, *p*>0.05<br>5-10 min: "group" effect, $F_{2,25}$=0.353, *p*>0.05<br>10-15 min: "group" effect, $F_{2,25}$=0.091, *p*>0.05
One-way ANOVA<br>(Traveling duration)	0-5 min: "group" effect $F_{2,25}$=0.903, *p*>0.05<br>5-10 min: "group" effect $F_{2,25}$=0.251, *p*>0.05<br>10-15 min: "group" effect $F_{2,25}$=0.032, *p*>0.05
One-way ANOVA<br>(Rearing duration)	0-5 min: "group" effect, $F_{2,25}$=0.345, *p*>0.05<br>5-10 min: "group" effect, $F_{2,25}$=0.398, *p*>0.05<br>10-15 min: "group" effect, $F_{2,25}$=0.522, *p*>0.05
One-way ANOVA<br>(Rearing count)	0-5 min: "group" effect, $F_{2,25}$=0.566, *p*>0.05<br>5-10 min: "group" effect, $F_{2,25}$=0.478, *p*>0.05<br>10-15 min: "group" effect, $F_{2,25}$=0.374, *p*>0.05
One-way ANOVA<br>(Thigmotaxis)	0-5 min: "group" effect, $F_{2,25}$=0.113, *p*>0.05<br>5-10 min: "group" effect, $F_{2,25}$=0.557, *p*>0.05<br>10-15 min: "group" effect = $F_{2,25}$=0.581, p>0.05
One-way ANOVA<br>(Center stay)	0-5 min: "group" effect, $F_{2,25}$=1.205, *p*>0.05<br>5-10 min: "group" effect, $F_{2,25}$=1.493, *p*>0.05<br>10-15 min: "group" effect, $F_{2,25}$=1.097, *p*>0.05
One-way ANOVA<br>(Center entry)	0-5 min: "group" effect, $F_{2,25}$=0.762, *p*>0.05<br>5-10 min: "group" effect, $F_{2,25}$=3.012, *p*>0.05<br>10-15 min: "group" effect, $F_{2,25}$=0.936, *p*>0.05
Rotarod	
Mixed two-way ANOVA<br>(Staying time)	"group" effect: $F_{2,25}$=0.329, *p*>0.05<br>"trial" effect: $F_{2,50}$=16.468, ***p*<0.001**<br>"group x trial" effect: $F_{4,50}$=0.096, *p*>0.05
Mixed two-way ANOVA<br>(Reaching speed)	"group" effect: $F_{2,25}$=0.389, *p*>0.05<br>"trial" effect: $F_{2,50}$=16.731, ***p*<0.001**<br>"group x trial" effect: $F_{4,50}$=0.099, *p*>0.05
One-way ANOVA<br>(Staying time)	trial 1: "group" effect, $F_{2,25}$=0.54, *p*>0.05<br>trial 2: "group" effect, $F_{2,25}$=0.274, *p*>0.05<br>trial 3: "group" effect, $F_{2,25}$=0.084, *p*>0.05
One-way ANOVA<br>(Reaching speed)	trial 1: "group" effect, $F_{2,25}$=0.551, *p*>0.05<br>trial 2: "group" effect, $F_{2,25}$=0.296, *p*>0.05<br>trial 3: "group" effect, $F_{2,25}$=0.162, *p*>0.05

Reciprocal social interaction	
One-way ANOVA<br>(Interaction time)	"group" effect: $F_{2,16}$=0.161, *p*>0.05
Three-chamber social test-sociability trial	
Mixed two-way ANOVA<br>(Exploration time)	"group" effect: $F_{2,20}$=0.157, *p*>0.05<br>"object" effect: $F_{1,20}$=36.063, ***p*<0.001**<br>"group x object" effect: $F_{2,20}$=0.149, *p*>0.05
Paired *t*-test within group<br>stranger mouse vs empty cup	control: t_7=4.053, ***p*=0.005**<br>vermis IV/V: t_6=3.947, ***p*=0.008**<br>vermis VI/VII: t_7=2.986, ***p*=0.02**
One sample *t*-test within group<br>index vs 0	control: t_7=4.43, **p=0.003**<br>vermis IV/V: t_6=6.478, **p=0.001**<br>vermis VI/VII: t_7=4.251, **p=0.004**
One-way ANOVA<br>(Total exploration time)	"group" effect: $F_{2,20}$=0.157, *p*>0.05
Three-chamber social test-social novelty trial	
Mixed two-way ANOVA<br>(Exploration time)	"group" effect: $F_{2,20}$=0.142, *p*>0.05<br>"object" effect: $F_{1,20}$=15.205, ***p*=0.001**<br>"group x object" effect: $F_{2,20}$=1.185, *p*>0.05
Paired *t*-test within group<br>old stranger vs novel stranger	control: t_7=-2.688, ***p*=0.031**<br>vermis IV/V: t_6=-3.08, ***p*=0.022**<br>vermis VI/VII: t_7=-1.041, *p*>0.05
One sample *t*-test within group<br>index vs 0	control: t_7=3.684, ***p*=0.008**<br>vermis IV/V: t_6=3.065, ***p*=0.022**<br>vermis VI/VII: t_7=1.593, *p*>0.05
One-way ANOVA<br>(Total exploration time)	"group" effect: $F_{2,20}$=0.142, *p*>0.05
Social recognition test-learning trial	
Mixed two-way ANOVA<br>(Exploration time)	"group" effect: $F_{2,21}$=0.449, *p*>0.05<br>"object" effect: $F_{1,21}$=39.115, ***p*<0.001**<br>"group x object" effect: $F_{1,21}$=0.307, *p*>0.05
Paired *t*-test within group<br>stranger mouse vs empty cup	control: t_7=3.522, ***p*=0.01**<br>vermis IV/V: t_7=3.989, ***p*=0.005**<br>vermis VI/VII: t_7=3.386, ***p*=0.012**
One sample *t*-test within group<br>index vs 0	control: t_7=6.62, ***p*<0.001**<br>vermis IV/V: t_7=8.388, ***p*<0.001**<br>vermis VI/VII: t_7=5.105, ***p*=0.001**
One-way ANOVA<br>(Total exploration time)	"group" effect: $F_{2,21}$=0.449, *p*>0.05
Social recognition test-testing trial	
Mixed two-way ANOVA<br>(Exploration time)	"group" effect: $F_{2,21}$=0.224, *p*>0.05<br>"object" effect: $F_{1,21}$=6.637, ***p*=0.018**<br>"group x object" effect: $F_{1,21}$=1.426, *p*>0.05
Paired *t*-test within group<br>old stranger vs novel stranger	control: t_7=-4.668, ***p*=0.002**<br>vermis IV/V: t_7=-0.899, *p*>0.05<br>vermis VI/VII: t_7=-0.588, *p*>0.05
One sample *t*-test within group	control: t_7=5.483, ***p*=0.001**

index vs 0	vermis IV/V: t_7=0.804, $p>0.05$<br>vermis VI/VII: t_7=0.114, $p>0.05$
One-way ANOVA<br>(Total exploration time)	“group” effect: $F_{2,21}$=0.224, $p>0.05$
Object recognition test-learning trial	
One-way ANOVA<br>(Total exploration time)	“group” effect: $F_{2,21}$=0.588, $p>0.05$
Object recognition test-testing trial	
Mixed two-way ANOVA<br>(Exploration time)	“group” effect: $F_{2,21}$=2.194, $p>0.05$<br>“object” effect: $F_{1,21}$=31.786, **$p<0.001$**<br>“group x object” effect: $F_{2,21}$=0.085, $p>0.05$
Paired *t*-test within group<br>old object vs novel object	control: t_7=-3.022, **p=0.019**<br>vermis IV/V: t_7=-3.544, **p=0.009**<br>vermis VI/VII: t_7=-3.393, **p=0.012**
One sample *t*-test within group<br>index vs 0	control: t_7=2.455, **p=0.044**<br>vermis IV/V: t_7=3.934, **p=0.006**<br>vermis VI/VII: t_7=3.952, **p=0.006**
One-way ANOVA<br>(Total exploration time)	“group” effect: $F_{2,21}$=2.194, $p>0.05$
**Chemogenetic manipulation – c-Fos imaging**	
One-way ANOVA<br>(c-Fos signals)	Acg: “group” effect, $F_{2,15}$=5.027, **p=0.021**<br>PL: “group” effect, $F_{2,15}$=4.389, **p=0.032**<br>IL: “group” effect: $F_{2,15}$=6.454, **p=0.009**<br>OFC: “group” effect, $F_{2,15}$=1.122, $p>0.05$<br>ACC: “group” effect, $F_{2,15}$=0.55, $p>0.05$<br>M1: “group” effect, $F_{2,15}$=3.066, $p>0.05$<br>dSTR: “group” effect, $F_{2,15}$=0.794, $p>0.05$<br>NAc: “group” effect, $F_{2,15}$=0.041, $p>0.05$<br>RSD: “group” effect, $F_{2,15}$=6.088, **p=0.012**<br>RSG: “group” effect, $F_{2,15}$=0.836, $p>0.05$<br>PtA: “group” effect, $F_{2,15}$=2.433, $p>0.05$<br>TeA: “group” effect, $F_{2,15}$=6.1, **p=0.012**<br>Ect: “group” effect, $F_{2,15}$=6.843, **p=0.008**<br>PRh: “group” effect, $F_{2,15}$=4.429, **p=0.031**<br>lEnt: “group” effect, $F_{2,15}$=1.228, $p>0.05$<br>CA1: “group” effect, $F_{2,15}$=0.025, $p>0.05$<br>CA2/3: “group” effect, $F_{2,15}$=0.295, $p>0.05$<br>DG: “group” effect, $F_{2,15}$=0.223, $p>0.05$<br>CeA: “group” effect, $F_{2,15}$=7.726, **p=0.005**<br>BLA: “group” effect, $F_{2,15}$=5.903, **p=0.013**<br>BMP: “group” effect, $F_{2,15}$=3.54, p=0.055<br>vTH: “group” effect, $F_{2,15}$=1.522, $p>0.05$<br>mHA: “group” effect, $F_{2,15}$=8.862, **p=0.003**<br>VTA: “group” effect, $F_{2,15}$=11.899, **p=0.001**
Post-hoc Fisher’s LSD test<br>(c-Fos signals)	Acg:<br>control vs vermis IV/V: **p=0.024**<br>vermis IV/V vs vermis VI/VII: **p=0.01**

	PL:<br>control vs vermis IV/V: ***p*=0.044**<br>vermis IV/V vs vermis VI/VII: ***p*=0.013**<br>IL:<br>control vs vermis VI/VII: ***p*=0.014**<br>vermis IV/V vs vermis VI/VII: ***p*=0.004**<br>RSD:<br>control vs vermis IV/V: ***p*=0.004**<br>vermis IV/V vs vermis VI/VII: ***p*=0.023**<br>TeA:<br>control vs vermis IV/V: ***p*=0.006**<br>control vs vermis VI/VII: ***p*=0.014**<br>Ect:<br>control vs vermis IV/V: ***p*=0.008**<br>control vs vermis VI/VII: ***p*=0.004**<br>PRh:<br>control vs vermis IV/V: ***p*=0.049**<br>control vs vermis VI/VII: ***p*=0.012**<br>CeA:<br>control vs vermis VI/VII: ***p*=0.003**<br>vermis IV/V vs vermis VI/VII: ***p*=0.006**<br>BLA:<br>control vs vermis VI/VII: ***p*=0.007**<br>vermis IV/V vs vermis VI/VII: ***p*=0.013**<br>mHA:<br>control vs vermis VI/VII: ***p*=0.009**<br>vermis IV/V vs vermis VI/VII: ***p*=0.001**<br>VTA:<br>control vs vermis IVI/V: ***p*=0.01**<br>vermis IV/V vs vermis VI/VII: ***p*<0.001**
One-way ANOVA<br>(Pearson correlation coefficient *r*; Fisher *Z* transformed)	All regions: "group" effect, $F_{2,1725}$=21.661, ***p*<0.001**<br>mPFC: "group" effect, $F_{2,213}$=4.492, ***p*=0.012**<br>ACC: "group" effect, $F_{2,69}$=9.625, ***p*<0.001**<br>Hippocampus: "group" effect, $F_{2,213}$=23.472, ***p*<0.001**<br>Amygdala: "group" effect, $F_{2,213}$=4.076, ***p*=0.018**
Post-hoc Fisher's LSD test<br>(Pearson correlation coefficient *r*; Fisher *Z* transformed)	All regions:<br>control vs vermis IV/V: ***p*<0.001**<br>control vs vermis VI/VII: ***p*=0.001**<br>mPFC:<br>control vs vermis IV/V: ***p*=0.006**<br>control vs vermis VI/VII: ***p*=0.021**<br>ACC:<br>control vs vermis IV/V: ***p*<0.001**<br>vermis IV/V vs vermis VI/VII: ***p*=0.01**<br>Hippocampus:<br>control vs vermis IV/V: ***p*<0.001**<br>control vs vermis VI/VII: ***p*<0.001**<br>Amygdala:

	control vs vermis VI/VII: ***p*=0.005**
**Optogenetic manipulation – electrophysiology**	
Paired *t*-test within group<br>light OFF vs light ON	MLIs: t_6=-3.232, ***p*=0.018**<br>PCs-frequency: t_8=3.794, ***p*=0.005**<br>PCs-CV: t_8=-8.419, ***p*<0.001**
**Optogenetic manipulation – behavioral tests**	
Social recognition test (light ON in learning trial)	
Learning trial	
Mixed two-way ANOVA<br>(Exploration time)	"group" effect: $F_{2,19}$=4.692, ***p*=0.022**<br>"object" effect: $F_{1,19}$=55.301, ***p*<0.001**<br>"group x object" effect: $F_{2,19}$=1.446, *p*>0.05
Paired *t*-test within group<br>stranger mouse vs empty cup	control: t_7=3.913, ***p*=0.006**<br>vermis IV/V: t_6=3.946, ***p*=0.008**<br>vermis VI/VII: t_6=5.904, ***p*=0.001**
One sample *t*-test within group<br>index vs 0	control: t_7=5.619, ***p*=0.001**<br>vermis IV/V: t_6=4.808, ***p*=0.003**<br>vermis VI/VII: t_6=7.244, ***p*<0.001**
One-way ANOVA<br>(Total exploration time)	"group" effect: $F_{2,19}$=4.692, ***p*=0.022**
Post-hoc Fisher's LSD test<br>(Total exploration time)	control vs vermis IV/V: ***p*=0.042**<br>vermis IV/V vs vermis VI/VII: ***p*=0.008**
Testing trial	
Mixed two-way ANOVA<br>(Exploration time)	"group" effect: $F_{2,19}$=10.918, ***p*=0.001**<br>"object" effect: $F_{1,19}$=22.39, ***p*<0.001**<br>"group x object" effect: $F_{2,19}$=1.341, *p*>0.05
Paired *t*-test within group<br>old stranger vs novel stranger	control: t_7=3.387, ***p*=0.012**<br>vermis IV/V: t_6=4.421, ***p*=0.004**<br>vermis VI/VII: t_6=2.571, ***p*=0.042**
One sample *t*-test within group<br>index vs 0	control: t_7=4.364, ***p*=0.003**<br>vermis IV/V: t_6=5.665, ***p*=0.001**<br>vermis VI/VII: t_6=2.6, ***p*=0.041**
One-way ANOVA<br>(Total exploration time)	"group" effect: $F_{2,19}$=10.918, ***p*=0.001**
Post-hoc Fisher's LSD test<br>(Total exploration time)	control vs vermis IV/V: ***p*=0.005**<br>vermis IV/V vs vermis VI/VII: ***p*<0.001**
Social recognition test (light ON in testing trial)	
Learning trial	
Mixed two-way ANOVA<br>(Exploration time)	"group" effect: $F_{2,22}$=1.593, *p*>0.05<br>"object" effect: $F_{1,22}$=42.403, ***p*<0.001**<br>"group x object" effect: $F_{2,22}$=0.134, *p*>0.05
Paired *t*-test within group<br>stranger mouse vs empty cup	control: t_8=5.903, ***p*<0.001**<br>vermis IV/V: t_7=4.404, ***p*=0.003**<br>vermis VI/VII: t_7=2.443, ***p*=0.045**
One sample *t*-test within group<br>index vs 0	control: t_8=6.435, ***p*<0.001**<br>vermis IV/V: t_7=5.714, ***p*=0.001**

	vermis VI/VII: t_7=2.032, *p*=0.052
One-way ANOVA<br>(Total exploration time)	"group" effect: $F_{2,22}$=1.596, *p*>0.05
Testing trial	
Mixed two-way ANOVA<br>(Exploration time)	"group" effect: $F_{2,22}$=3.352, *p*>0.05<br>"object" effect: $F_{1,22}$=1.993, *p*>0.05<br>"group x object" effect: $F_{2,22}$=7.828, **p=0.003**
Paired *t*-test within group<br>old stranger vs novel stranger	control: t_8=3.539, ***p*=0.008**<br>vermis IV/V: t_7=0.277, *p*>0.05<br>vermis VI/VII: t_7=1.233, *p*>0.05
One sample *t*-test within group<br>index vs 0	control: t_8=4.222, ***p*=0.003**<br>vermis IV/V: t_7=0.361, *p*>0.05<br>vermis VI/VII: t_7=0.426, *p*>0.05
One-way ANOVA<br>(Total exploration time)	"group" effect: $F_{2,22}$=3.352, *p*>0.05
Object recognition test (light ON in testing trial)	
Mixed two-way ANOVA<br>(Exploration time)	"group" effect: $F_{2,23}$=0.086, *p*>0.05<br>"object" effect: $F_{1,23}$=18.525, ***p*<0.001**<br>"group x object" effect: $F_{2,23}$=0.005, *p*>0.05
Paired *t*-test within group<br>old object vs novel object	control: t_8=2.582, **p=0.033**<br>vermis IV/V: t_7=2.343, *p*=0.052<br>vermis VI/VII: t_8=2.538, ***p*=0.035**
One sample *t*-test within group<br>index vs 0	control: t_8=3.805, ***p*=0.005**<br>vermis IV/V: t_7=3.707, ***p*=0.008**<br>vermis VI/VII: t_8=4.089, ***p*=0.003**
One-way ANOVA<br>(Total exploration time)	Learning trial:<br>"group" effect: $F_{2,23}$=2.786, *p*>0.05<br>Testing trial:<br>"group" effect: $F_{2,23}$=0.086, *p*>0.05

Note: All *t*-tests and Fisher's LSD tests were two-tailed tests.
