# Supplementary material for: Social memory deficit caused by dysregulation of the cerebellar vermis
Source: Nat Commun. 2023 Sep 26;14:6007. doi: 10.1038/s41467-023-41744-2 (PMC10522595; doi:10.1038/s41467-023-41744-2)
Supplement: Supplementary file 3 — Reporting Summary [file 41467_2023_41744_MOESM3_ESM.pdf]

nature portfolio

Corresponding author(s): Yi-Mei Yang

Last updated by author(s): Jun 30, 2023

Reporting Summary

Nature Portfolio wishes to improve the reproducibility of the work that we publish. This form provides structure for consistency and transparency in reporting. For further information on Nature Portfolio policies, see our Editorial Policies and the Editorial Policy Checklist.

Statistics

For all statistical analyses, confirm that the following items are present in the figure legend, table legend, main text, or Methods section.

n/a	Confirmed	
☐	☒	The exact sample size (*n*) for each experimental group/condition, given as a discrete number and unit of measurement
☐	☒	A statement on whether measurements were taken from distinct samples or whether the same sample was measured repeatedly
☐	☒	The statistical test(s) used AND whether they are one- or two-sided<br>*Only common tests should be described solely by name; describe more complex techniques in the Methods section.*
☒	☐	A description of all covariates tested
☐	☒	A description of any assumptions or corrections, such as tests of normality and adjustment for multiple comparisons
☐	☒	A full description of the statistical parameters including central tendency (e.g. means) or other basic estimates (e.g. regression coefficient) AND variation (e.g. standard deviation) or associated estimates of uncertainty (e.g. confidence intervals)
☐	☒	For null hypothesis testing, the test statistic (e.g. *F*, *t*, *r*) with confidence intervals, effect sizes, degrees of freedom and *P* value noted<br>*Give P values as exact values whenever suitable.*
☒	☐	For Bayesian analysis, information on the choice of priors and Markov chain Monte Carlo settings
☒	☐	For hierarchical and complex designs, identification of the appropriate level for tests and full reporting of outcomes
☐	☒	Estimates of effect sizes (e.g. Cohen's *d*, Pearson's *r*), indicating how they were calculated

Our web collection on statistics for biologists contains articles on many of the points above.

Software and code

Policy information about availability of computer code

Data collection	Electrophysiological data were acquired using a MultiClamp 700B amplifier (Molecular Devices) and a Digidata 1550B digitizer (Molecular Devices) supported by pCLAMP 10 software (Molecular Devices). Mouse behavioral data were collected using ANY-maze tracking software (Stoelting). Confocal images were taken using a Zeiss LSM 710 confocal microscope and Zen 2.0 software.
Data analysis	Electrophysiological data were analyzed using MiniAnalysis 6.0.7 (Synaptosoft), Clampfit 10 (Molecular Devices) and Excel 2016 (Microsoft). Mouse behavioral data were analyzed using ANY-maze software (Stoelting). Imaging data were analyzed using ImageJ (NIH). Graph theoretical analysis was performed using MATLAB (R2015b) with Brain Connectivity Toolbox (https://sites.google.com/site/bctnet/). Statistical analysis was performed using IBM SPSS statistics 25 (IBM Corp) and GraphPad Prism 9.3.1 (GraphPad Software).

For manuscripts utilizing custom algorithms or software that are central to the research but not yet described in published literature, software must be made available to editors and reviewers. We strongly encourage code deposition in a community repository (e.g. GitHub). See the Nature Portfolio guidelines for submitting code & software for further information.

Data

Policy information about availability of data

All manuscripts must include a data availability statement. This statement should provide the following information, where applicable:

- Accession codes, unique identifiers, or web links for publicly available datasets
- A description of any restrictions on data availability
- For clinical datasets or third party data, please ensure that the statement adheres to our policy

All data are available in the main text and supplementary information. Source data are provided with this paper and deposited to the Figshare (https://doi.org/10.6084/m9.figshare.22647700). The mouse brain atlas used in this study is publicly available.

Research involving human participants, their data, or biological material

Policy information about studies with human participants or human data. See also policy information about sex, gender (identity/presentation), and sexual orientation and race, ethnicity and racism.

Reporting on sex and gender	Not applicable.
Reporting on race, ethnicity, or other socially relevant groupings	Not applicable.
Population characteristics	Not applicable.
Recruitment	Not applicable.
Ethics oversight	Not applicable.

Note that full information on the approval of the study protocol must also be provided in the manuscript.

Field-specific reporting

Please select the one below that is the best fit for your research. If you are not sure, read the appropriate sections before making your selection.

☒ Life sciences ☐ Behavioural & social sciences ☐ Ecological, evolutionary & environmental sciences

For a reference copy of the document with all sections, see nature.com/documents/nr-reporting-summary-flat.pdf

Life sciences study design

All studies must disclose on these points even when the disclosure is negative.

Sample size	No statistical methods were used to predetermine sample sizes. The sample sizes were based on previous studies using similar experimental protocols (PMID: 33661502; 32179875; 30224722): 8-12 mice/group for behavioral tests, 6-10 cells from >3 mice for electrophysiological recordings, 3-5 mice for anatomical studies. The number of samples was selected to ensure valid conclusions while following the ethical guidelines for the use of laboratory animals. The sample sizes were considered sufficient due to low observed variabilities between samples.
Data exclusions	To control the quality of electrophysiology recordings, we only included cells in good health with clearly identifiable structure, and recordings with >1 GΩ seal resistance in the cell-attached mode. To control the quality of stereotactic surgery, we excluded mice with injections outside the intended areas. To control the quality of behavioral testing, we excluded mice that lacked exploration or showed a bias for a certain place during habituation. These criteria were in line with common practices in the field to enhance reliability and validity of the results.
Replication	Each experiment under the same condition was repeated in a number of cells and animals to control individual variabilities. All measurements were taken from distinct samples. The sample size for each group was stated in the Results, Methods, Figure Legends, Source Data, and Supplementary Information. All attempts at replication were successful. For example, the major finding of this study, i.e., a social memory deficit caused by cerebellar perturbation, was replicated in two independent sets of experiments using chemo- or optogenetic manipulation (Fig. 1 & 2).
Randomization	Mice of either sex were assigned to each group without discrimination for all experiments. The order in which they were picked was random. Neurons in cerebellar slices were identified by their morphology and location, and selected randomly for electrophysiological recording.
Blinding	Experimenters who performed the tests were not blinded to mouse genotypes because all experiments required the knowledge of genotypes to assign mutant mice and their wild-type littermates to certain groups. However, all data analyses were done by different experimenters who were blinded to the experimental design.

Reporting for specific materials, systems and methods

We require information from authors about some types of materials, experimental systems and methods used in many studies. Here, indicate whether each material, system or method listed is relevant to your study. If you are not sure if a list item applies to your research, read the appropriate section before selecting a response.

Materials & experimental systems

n/a	Involved in the study
☐	☒ Antibodies
☒	☐ Eukaryotic cell lines
☒	☐ Palaeontology and archaeology
☐	☒ Animals and other organisms
☒	☐ Clinical data
☒	☐ Dual use research of concern
☒	☐ Plants

Methods

n/a	Involved in the study
☒	☐ ChIP-seq
☒	☐ Flow cytometry
☒	☐ MRI-based neuroimaging

Antibodies

Antibodies used

Primary antibodies:
(1) rabbit anti-Calbindin D28K (Invitrogen, PA1-931, lot # SE249690, dilution 1:1000)
(2) rabbit anti-CaMKII (Invitrogen, PA5-99558, lot # VH3057604, dilution 1:200)
(3) mouse anti-GAD67 (Millipore, MAB5406, lot # 3091709, clone # 1G10.2, dilution 1:500)
(4) guinea pig anti-c-Fos (Synaptic Systems, 226308, lot # 1-5, dilution 1:1000)
(5) rabbit anti-c-Fos (Abcam, ab190289, lot # GR3185049-2, dilution 1:4000)

Secondary antibodies:
(1) goat anti-rabbit Cy5 (Invitrogen; A10523, lot # 1843847, dilution 1:500)
(2) goat anti-guinea pig Alexa 488 (Invitrogen, A11073, lot # 1841755, dilution 1:1000)
(3) goat anti-rabbit Alexa 555 (Invitrogen, A21428, lot # 2445825, dilution 1:1000)
(4) goat anti-mouse Alexa 647 (Invitrogen, A21236, lot # 2482947,dilution 1:1000)

Validation

All primary antibodies were recombinant peptides produced using in vitro expression systems, which provides better specificity and sensitivity, lot-to-lot consistency, animal origin-free formulations, and broader immunoreactivity to diverse targets. All antibodies were validated by their manufacturers and optimized for use in the lab.

(1) rabbit anti-Calbindin D28K antibody was validated using Western blot in tissue lysate (mouse/rat brain), and immunocytochemistry in PC12 cells (https://www.thermofisher.com/antibody/product/Calbindin-D28K-Antibody-Polyclonal/PA1-931). Citations include: PMID: 34803580; 34165156.

(2) rabbit anti-CaMKII antibody was validated using Western blot in mouse tissue, and immunohistochemistry in paraffin-embedded mouse issue (https://www.thermofisher.com/antibody/product/CaMKII-alpha-beta-delta-Antibody-Polyclonal/PA5-99558). Citations include: PMID:31972088.

(3) mouse anti-GAD67 antibody was validated using immunohistochemistry in PFA-fixed mouse/rat tissue (https://www.sigmaaldrich.com/US/en/product/mm/mab5406). Independently, we have verified the antibodies in our previous publications: PMID: 33661502; 32179875.

(4) guinea pig anti-c-Fos antibody (Synaptic Systems) was validated using Western blot in HeLa cell lysate, and immunohistochemistry in paraffin-embedded mouse tissue (https://sysy.com/product/226308). Citations include: PMID: 36990091; 35961989; 36061821.

(5) rabbit anti-c-Fos antibody (Abcam) was validated using Western blot in HeLa cell lysate, and immunohistochemistry in paraffin-embedded mouse tissue (https://www.abcam.com/c-fos-antibody-bsa-free-ab190289). Citations include: PMID: 36322337; 36323255; 36358391; 36516774.

Animals and other research organisms

Policy information about [studies involving animals](); [ARRIVE guidelines]() recommended for reporting animal research, and [Sex and Gender in Research]()

Laboratory animals

C-kit-Cre and nNOS-ChR2 BAC mice were provided by Hiroki Taniguchi (Ohio State University, USA) and George J Augustine (Nanyang Technological University, Singapore), respectively. Ai9 mice (strain # 007909) were purchased from the Jackson Laboratory. Heterozygous mice were bred to obtain mutants and littermate controls. Both male and female mice aged 8-12 weeks were used. Mice were kept under a 12-hour light-dark cycle (light on from 07:00 to 19:00) and reared 3-5 per cage with food and water ad libitum. The ambient temperature and humidity were 22-26 Celsius and 40-70%.

Wild animals

No wild animals were used in this study.

Reporting on sex	Sex as a biological variable was considered in the study design. Both male and female animals (in comparable numbers) were included in all groups. No sex differences in the results were observed.
Field-collected samples	No field-collected samples were used in this study.
Ethics oversight	All procedures were approved by the Institutional Animal Care and Use Committee (IACUC) and the Institutional Biosafety Committee (IBC) of University of Minnesota, in accordance with the National Institutes of Health guidelines.

Note that full information on the approval of the study protocol must also be provided in the manuscript.
